# *Escherichia coli* α-Hemolysin Counteracts the Anti-Virulence Innate Immune Response Triggered by the Rho GTPase Activating Toxin CNF1 during Bacteremia

**DOI:** 10.1371/journal.ppat.1004732

**Published:** 2015-03-17

**Authors:** Mamady Diabate, Patrick Munro, Elsa Garcia, Arnaud Jacquel, Gregory Michel, Sandrine Obba, Diogo Goncalves, Carmelo Luci, Sandrine Marchetti, Dieter Demon, Clara Degos, Yassina Bechah, Jean-Louis Mege, Mohamed Lamkanfi, Patrick Auberger, Jean-Pierre Gorvel, Lynda Maria Stuart, Luce Landraud, Emmanuel Lemichez, Laurent Boyer

**Affiliations:** 1 INSERM, U1065, Centre Méditerranéen de Médecine Moléculaire, C3M, Toxines Microbiennes dans la relation hôte pathogènes, Nice, France; 2 Université de Nice-Sophia-Antipolis, UFR Médecine, Nice, France; 3 Laboratoire de Bactériologie, CHU de Nice, Hôpital l’Archet, Nice, France; 4 INSERM, U1065, Centre Méditerranéen de Médecine Moléculaire, C3M, Mort Cellulaire, Differentiation et Cancer, Nice, France; 5 CNRS UMR7275, IPMC, Sophia Antipolis, France; 6 Department of Medical Protein Research, VIB, Ghent, Belgium; Department of Biochemistry, Ghent University, Ghent, Belgium; 7 Aix-Marseille University UM 2, INSERM U 1104, CNRS UMR 7280, Marseille, France; 8 Unité de Recherche sur les Maladies Infectieuses Tropicales et Emergentes, CNRS UMR 6236, Faculté de Médecine, Marseille, France; 9 Benaroya Research Institute at Virginia Mason, Seattle, Washington, United States of America; Northwestern University, Feinberg School of Medicine, UNITED STATES

## Abstract

The detection of the activities of pathogen-encoded virulence factors by the innate immune system has emerged as a new paradigm of pathogen recognition. Much remains to be determined with regard to the molecular and cellular components contributing to this defense mechanism in mammals and importance during infection. Here, we reveal the central role of the IL-1β signaling axis and Gr1+ cells in controlling the *Escherichia coli* burden in the blood in response to the sensing of the Rho GTPase-activating toxin CNF1. Consistently, this innate immune response is abrogated in caspase-1/11-impaired mice or following the treatment of infected mice with an IL-1β antagonist. *In vitro* experiments further revealed the synergistic effects of CNF1 and LPS in promoting the maturation/secretion of IL-1β and establishing the roles of Rac, ASC and caspase-1 in this pathway. Furthermore, we found that the α-hemolysin toxin inhibits IL-1β secretion without affecting the recruitment of Gr1+ cells. Here, we report the first example of anti-virulence-triggered immunity counteracted by a pore-forming toxin during bacteremia.

## Author Summary

The pathogenic potentials of most microbes depend on a repertoire of virulence factors. Despite major progress in the understanding of the molecular mechanisms underlying the activities of bacterial effectors, little is known about how they cooperate during infection to overcome host immune defenses and promote microbial persistence. Here, we investigated the roles of two uropathogenic *Escherichia coli* (UPEC) effectors that are co-ordinately expressed, α-hemolysin (HlyA) and cytotoxic necrotizing factor 1 (CNF1). We demonstrated that the HlyA toxin is critical for bacterial stability in the blood and showed that one important role of HlyA is to inhibit the CNF1-induced host response. Collectively, these findings reveal why the coordinated activities of HlyA and CNF1 are necessary for the full virulence of UPEC. Moreover, they unravel a HlyA-driven counter-defense mechanism used by bacteria to facilitate their survival.

## Introduction

Bacteremia caused by extraintestinal strains of pathogenic *Escherichia coli* is a leading cause of death worldwide [1,2]. Among these pathogens, uropathogenic *E*. *coli* (UPEC) is a major etiological agent of bacteremia [1,2]. Therefore, it is essential to define the mechanisms by which virulence factors of UPEC and innate immune signaling pathways control the bacterial burden in the blood.

The major virulence factors of UPEC have been characterized at the molecular level [3–6]. These factors include the presence of a specialized adhesive appendage and specific metabolic pathways as well as protein toxins; together, these features enable UPEC to efficiently colonize the urinary tract and translocate into the bloodstream of the host [7–9]. Two highly prevalent bacterial toxins, α-hemolysin (HlyA) and cytotoxic necrotizing factor-1 (CNF1), work together to damage and disrupt the cohesion of the uroepithelium, which additionally leads to the worsening of the host inflammatory reaction [10–12]. The high prevalences of *hlyA* and *cnf1* in uroseptic strains of UPEC suggest the possible functions of both of these toxins during bacteremia [9,13,14]. In contrast, it has not been determined whether both toxins contribute to the pathogen burden during bacteremia. HlyA belongs to a group of pore-forming leukotoxins that contain RTX repeats [13–15]. Depending on its concentration and on the type of cell intoxicated, HlyA either displays cytolytic activity or hijacks innate immune signaling pathways [13,16–18]. However, its role during bacteremia remains to be determined. The gene encoding the CNF1 toxin is located downstream from the α-hemolysin operon and is co-expressed with HlyA [19,20]. All CNF1-positive uroseptic strains display a hemolytic phenotype [9]. The CNF1 toxin possesses an enzymatic activity that is responsible for the posttranslational deamidation of a specific glutamine residue in a subset of small Rho GTPases, namely, Rac, Cdc42 and RhoA [21]. This type of modification increases the flux of activated Rho proteins and augments signaling through their downstream signaling pathways [21].

The activation of small Rho GTPases by virulence factors is a common trait of various enteric and extraintestinal Gram-negative pathogens. This activation of Rho GTPases confers upon bacteria the property to invade epithelial cells and tissues as well as to hijack inflammatory cell responses [22–25]. Emerging studies have indicated that cells are capable of perceiving the abnormal activation of Rac/Cdc42 induced by virulence factors of pathogens and translating this information via NOD1 and RIP1/RIP2 kinase signaling pathways into danger signals [26,27]. This innate immune mechanism involving the sensing of pathogens is here referred to as anti-virulence immunity (AVI), and it shares similarities with effector-triggered immunity (ETI), the mechanism by which plants sense the activities of bacterial effectors [28,29]. It will be important to define whether and how AVI triggers pathogen destruction in collaboration with the recognition of conserved microbial-associated patterns by pattern recognition receptors (PRR).

Inflammatory caspases, such as caspase-1 and caspase-11, drive innate immune responses to a variety of bacterial stimuli, such as microbe-associated molecular patterns (MAMPs) [e.g., lipopolysaccharide (LPS) or muramyl dipeptide (MDP)], as well as toxin-driven membrane damage [30]. Pathogen perception by NOD-like receptors triggers the assembly of inflammatory caspases in an operational ASC-dependent inflammasome complex that carries out the processing and release of the pro-inflammatory cytokine IL-1β [31,32]. The activation of inflammatory caspases by various type III injected effectors of *Salmonella*, notably those activating the small GTPase Rac, largely accounts for the induction of inflammatory responses triggered by enteric epithelial cells [33–35]. The means by which pathogenic bacteria overcome inflammatory responses, notably those driven by caspases, and succeed in infecting their hosts largely remains to be elucidated.

Here, we investigate the manner by which virulence factors of UPEC and innate immune signaling pathways impact the outcome of bacteremia. To this end, we focused on the role of CNF1 and HlyA, two toxins produced by UPEC, on pathogen burden in the bloodstream and on animal survival.

## Results

### CNF1 activity decreases pathogen load and favors host survival during bacteremia

**Fig 1:**
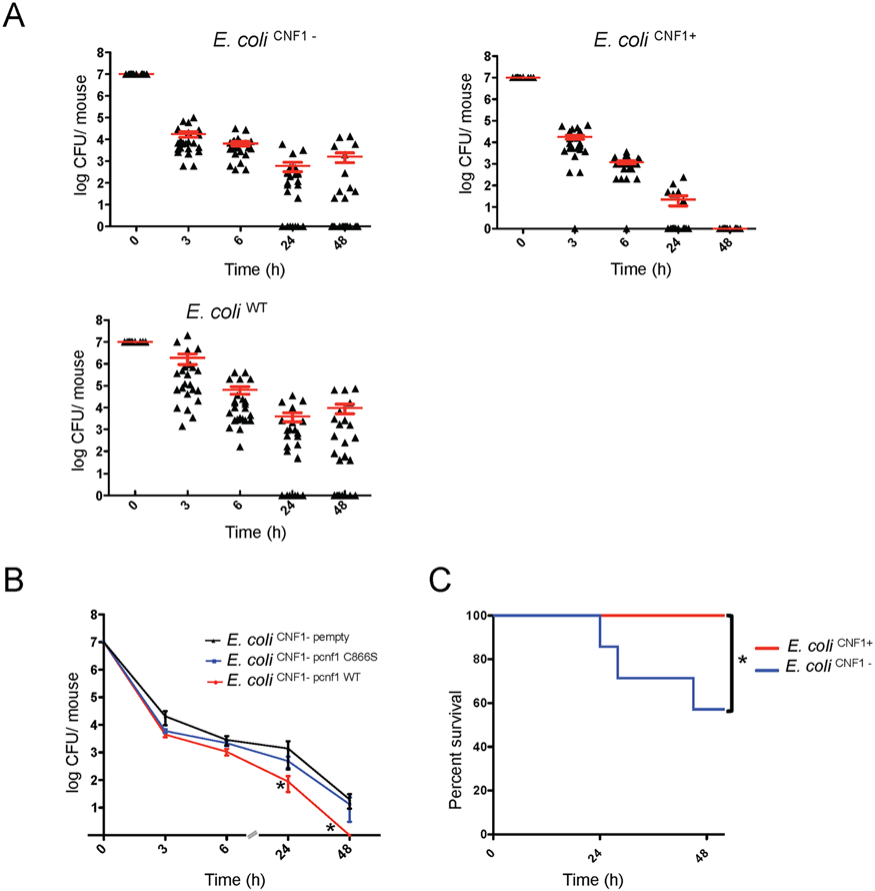
Infection with *E*. *coli* encoding CNF1 triggers clearance of bacteria from blood and mouse survival. (A) Female BALB/c mice were intravenously infected with 10^7^ CFUs of *E*. *coli* expressing CNF1, *E*. *coli* UTI89Δ*hlyA* (*E*. *coli* ^CNF1+^), or with the isogenic mutant UTI89Δ*hlyA*/Δ*cnf1* (*E*. *coli* ^CNF1-^) or the parental strain UTI89 (*E*. *coli* ^UTI89^) prior to the collection of peripheral blood at 3, 6, 24 and 48 h for the measurement of bacteremia (n = 25–30). The red line indicates the mean values. (B) Female BALB/c mice were intravenously infected with 10^7^ CFUs of the *E*. *coli* ^CNF1-^ strain transformed with a control empty plasmid (*E*. *coli* ^CNF1- pempty^), with a plasmid encoding the CNF1-inactive mutant C866S (*E*. *coli* ^CNF1- pcnf1 C866S^) or with a plasmid encoding *E*. *coli* CNF1 WT (*E*. *coli* ^CNF1-pcnf1 WT^) prior to the collection of peripheral blood at 3, 6, 24 and 48 h for the measurement of bacteremia (n = 6–12). For both (A) and (B), the data are expressed as the mean ± SEM (n = 6–30) at a *p<0.05. (C) BALB/c mouse survival was monitored for 52 h after intravenous injection of 2.10^8^ CFUs of *E*. *coli* ^CNF1+^ or the isogenic mutant, *E*. *coli* ^CNF1-^ (n = 20). *p<0.05 using the Gehan-Breslow-Wilcoxon chi-squared test.

We first assessed the role of the CNF1 toxin in determining *E*. *coli* burden during the course of bacteremia in the absence of interference from the other toxin, HlyA. For this purpose, we generated both an *hlyA* ^*-*^ deletion mutant (referred to as *E*. *coli* ^CNF1+^) and an *hlyA* ^*-*^ *cnf1* ^*-*^ double deletion mutant (referred to as *E*. *coli* ^CNF1-^) from *E*. *coli* ^WT^ UTI89. By characterizing the strains at the genetic and functional levels, we determined that the two mutants and the wild-type strain had identical growth properties (S1 and S2 Figs). BALB/c mice were then infected intravenously with *E*. *coli* ^CNF1+^ or *E*. *coli* ^CNF1-^ isogenic strains, and the pathogen load was monitored by the serial dilution of blood samples and the enumeration of CFUs (Fig. 1A). We found that the kinetics of clearance from the bloodstream of the *E*. *coli* ^CNF1+^ strain was very different (Fig. 1A). The *E*. *coli* ^CNF1+^ strain was rapidly cleared, with no bacteria detectable at 48 h p.i. compared with *E*. *coli* ^WT^ and *E*. *coli* ^CNF1-^, which produced 10^4^ and 10^3^ CFU/mouse, respectively, at 48 h p.i. (Fig. 1A). We next assessed whether the rapid clearance of the *E*. *coli* ^CNF1+^ strain was actually due to the enzymatic activity of CNF1. We tested this hypothesis by complementing the *E*. *coli* ^CNF1-^ strain with either an expression vector of wild-type CNF1 (*E*. *coli* ^CNF1- pcnf1^) or an expression vector of the catalytically inactive mutant CNF1 C866S (*E*. *coli* ^CNF1- pcnf1 C866S^) (Fig. 1B). We found that *E*. *coli* ^CNF1- pcnf1^ bacteria were cleared more rapidly from the blood than *E*. *coli* ^CNF1- pcnf1 C866S^ (Fig. 1B). Together, these results indicate that CNF1 activity promotes the eradication of bacteria from the bloodstream.

To discern whether there is a link between the effects of CNF1 on pathogen burden and strain virulence, we monitored the deaths of infected animals. To this end, *E*. *coli* ^CNF1-^ bacteria were injected at a dose sufficient to kill half of the mice by 48 h p.i. Animal survival following injection with the different isogenic mutants was monitored (Fig. 1C). We found that all of the mice infected with *E*. *coli* ^CNF1+^ survived, whereas the group of mice infected with *E*. *coli* ^CNF1-^ displayed only 57% survival (Fig. 1C).

Taken together, our data establish that CNF1 activity has a detrimental effect on the bacterial burden in the blood and that it protects against pathogen-induced animal death.

### CNF1 potentiates the LPS-triggered secretion of IL-1β in an inflammatory caspase-dependent manner

**Fig 2:**
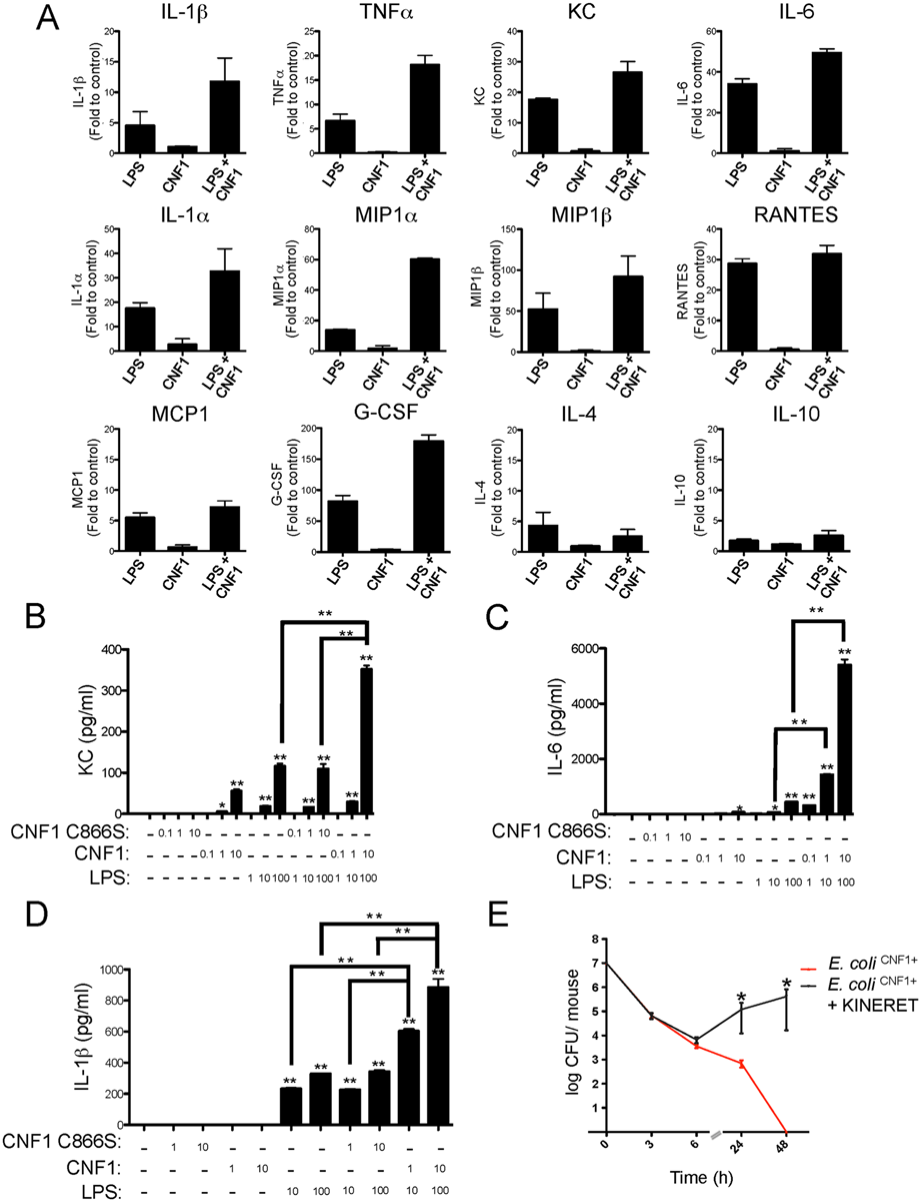
CNF1 potentiates LPS-triggered immune responses. (A) Monocytes (5x10^5^ cells per condition) isolated from mouse blood were treated with PBS (control) or with 1 μg/ml CNF1 toxin for 10 h with or without 100 ng/ml LPS. Cell culture supernatants were analyzed using mouse ELISArray kits (n = 3). The data are shown as fold inductions compared with the control condition. (B, C and D) Monocytes (5x10^5^ cells per condition) purified from mouse blood were treated for 10 h. Cells were treated as indicated with 0.1, 1, or 10 μg/ml of CNF1 toxin or the CNF1 mutant C866S alone or in combination with ultrapure *E*. *coli* LPS at 1, 10 or 100 ng/ml (n = 3). (B) KC, (C) IL-6, and (D) IL-1β cytokine secretion was analyzed using ELISA (n = 4). (E) Female BALB/c mice were intravenously infected with 10^7^ CFUs of *E*. *coli* expressing CNF1 (*E*. *coli* ^CNF1+^) + PBS as a control or with an *E*. *coli* ^CNF1+^ + IL-1β antagonist (Kineret; 1.5 mg/kg) prior to the collection of peripheral blood at 3, 6, 24 and 48 h for the measurement of bacteremia (n = 10). P-values <0.05 (*); and P-values <0.01 (**) were considered statistically significant.

We hypothesized that CNF1 activity has a negative impact on bacterial burden via the modulation of LPS-driven antimicrobial host responses. We assessed this conjecture by profiling the cytokines and chemokines secreted by primary monocytes isolated from the blood of mice after various experimental treatments. The monocytes were challenged with ultrapure LPS, with CNF1 alone, or with a combination of both factors. We used an unbiased approach that utilized an ELISArray semi-quantitative cytokine/chemokine screen to measure the levels of the following factors: IL-1β, TNFα, KC, IL-6, IL-1α, MIP1α, MIP1β, RANTES, MCP1, IL-12, MDC, MIG, IL17, IP10, TARC, EOTAXIN, IL-2, IL-4, IL-5, IL-10, IL-13, IL-23, INFγ, TNFβ1, GM-CSF, and G-CSF. Fig. 2A shows the panel of cytokines that were synergistically induced by LPS+CNF1 compared to ultrapure LPS or CNF1 alone. The results are presented as fold inductions compared to untreated monocytes for each treatment condition Figs. (2A and S3). The panel of cytokines produced by the monocytes treated with CNF1 alone is presented (S4A Fig). Other molecular mediators, such as the IL-4 and IL-10 anti-inflammatory cytokines, showed no significant induction in the monocytes treated with LPS+CNF1 (Fig. 2A). In support of our *in vitro* analysis results, we measured higher increases in these inflammatory mediators in the sera of mice infected with *E*. *coli* ^CNF1+^compared with *E*. *coli* ^CNF1-^ (S4B Fig). Collectively, these results show that CNF1 activity potentiates the LPS-triggered secretion of the pro-inflammatory cytokines IL-1β, TNFα, and IL-6 primarily, as well as the secretion of the chemokines MCP1, MIP1α, MIP1β, and KC.

We next performed quantitative analysis of the impact of CNF1 activity on the monocyte responses to LPS. Primary monocytes isolated from the blood of naïve mice were treated with endotoxin-free CNF1 or with the catalytically inactive mutant CNF1 C866S. In the monocytes treated with recombinant purified CNF1, we observed the production of KC (75 +/- 5 pg/ml), which was strictly dependent upon the activity of CNF1 (Fig. 2B). Control experiments with ultrapure LPS alone or in combination with the catalytically inactive mutant CNF1 C866S triggered the secretion of KC (120 +/-10 pg/ml). Strikingly, we observed a 3-fold increase in the production of KC (350 +/-10 pg/ml) in the cells treated with both LPS and CNF1 compared with those treated with ultrapure LPS alone (Fig. 2B). The co-stimulation of monocytes with CNF1 and LPS resulted in a 12-fold increase in IL-6 secretion and a 2-fold increase in IL-1β secretion compared with stimulation with LPS alone (Fig. 2C and D). This synergy was detected with doses of CNF1 as low as 10 ng/ml and was of the same magnitude as that observed with the ATP treatment (S4C Fig). We also observed that CNF1 acts synergistically with other Toll-like receptors (TLR) ligands, such as Pam3CSK4 (TLR1/TLR2 agonist) and FSL-1 (TLR2/TLR6 agonist) (S4D Fig). IL-1β is an important mediator of inflammatory responses and is notably important in enabling the host to mount an efficient antibacterial immune response [36,37]. As a first approach, to evaluate the role of IL-1β in the elimination of pathogens, we treated infected mice with an IL-1β antagonist (KINERET). We found that this treatment dramatically antagonized the clearance of *E*. *coli* ^CNF1+^ (Fig. 2E).

Collectively, our data pointed for the importance of the synergic induction of IL-1β by LPS+CNF1 in promoting efficient bacterial clearance from the bloodstream.

### CNF1 anti-virulence immunity is mediated by caspase-1

**Fig 3:**
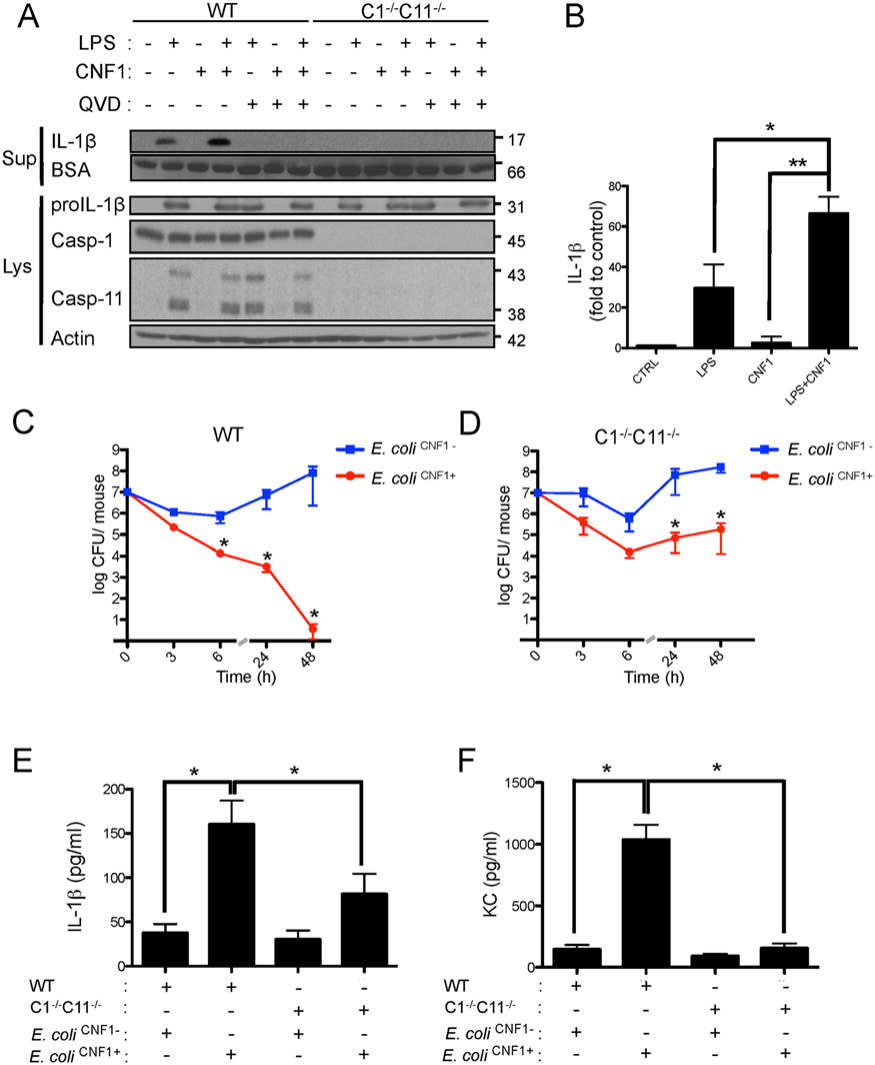
CNF1-triggered immunity requires inflammatory caspases. (A and B) IL-1β production and maturation/secretion after treatment with CNF1, LPS or CNF1 (1 μg/ml) + LPS (100 ng/ml) for 10 h. Actin and BSA were used as loading controls. (B) Graph showing the quantification of IL-1β secretion normalized to the control (n = 3). (C and D) Female C57BL/6 WT (C) or congenic C1^-/-^C11^-/-^ mice (D) were intravenously infected with 10^7^ CFUs of *E*. *coli* ^CNF1+^ or with the isogenic CNF1-deleted mutant (*E*. *coli* ^CNF1-^) prior to the collection of peripheral blood at 3, 6, 24 and 48 h for bacteremia measurements. The data are expressed as the mean ± SEM (n = 7–8). (E and F) Analysis of the circulating levels of IL-1β (E) and KC (F) in the mouse sera by ELISA. Serum samples from mice infected with 10^7^ *E*. *coli* ^CNF1+^ or with the isogenic mutant *E*. *coli* ^CNF1-^ were collected at 3 h after intravenous infection and analyzed by ELISA (n = 3). P-values<0.05 (*); and P-values<0.01 (**) were considered statistically significant.

Given the critical role of IL-1β signaling in the elimination of bacteria, we next aimed to precisely determine the components required for IL-1β maturation. IL-1β is expressed as a proform (proIL-1β) that is processed by caspases-1/11 to generate the mature p17-secreted active form [38,39]. We further assessed the effects of the interplay between LPS and CNF1 on IL-1β maturation. This interaction was evaluated by immunoblotting to determine the level of secretion of p17 IL-1β into the medium of monocytes upon co-stimulation with LPS+CNF1 (Fig. 3A and B). Our results confirmed that CNF1 acts at the level of IL-1β maturation/secretion rather than at the level of IL-1β expression (Fig. 3A and B). We observed the complete inhibition of the release of p17 IL-1β in the monocytes treated with the pan-caspase inhibitor QVD as well as in the monocytes isolated from caspase-1/11 (C1-C11)-impaired mice (Fig. 3A and B). Notably, these results indicate that CNF1 plays a critical role in promoting the caspase-1/11-dependent maturation/secretion of IL-1β by monocytes challenged with LPS. We next assessed the interplay between inflammatory caspases and CNF1 during UPEC-induced bacteremia. To this end, we measured bacterial loads in the blood of C1-C11-impaired mice infected with *E*. *coli* ^CNF1+^ or *E*. *coli* ^CNF1-^. We compared the kinetics of the bacterial burden in these animals with those of their wild-type congenic C57BL/6 littermates. In the wild-type animals, we measured a decrease in the bacterial load in the animals infected with *E*. *coli* ^CNF1+^, with no bacteria detectable in the blood at 48 h, compared with the presence of 10^7^ CFU/animal in the blood of the mice infected with *E*. *coli* ^CNF1-^ (Fig. 3C). In contrast with the wild-type mice, the *E*. *coli* ^CNF1+^ burden in the C1-C11-impaired mice remained high, reaching 10^5^ CFU/animal at 48 h p.i. (Fig. 3D). These results indicate that inflammatory caspases-1/11 play major roles in the clearance of bacteria from the bloodstream as triggered by CNF1.

**Fig 4:**
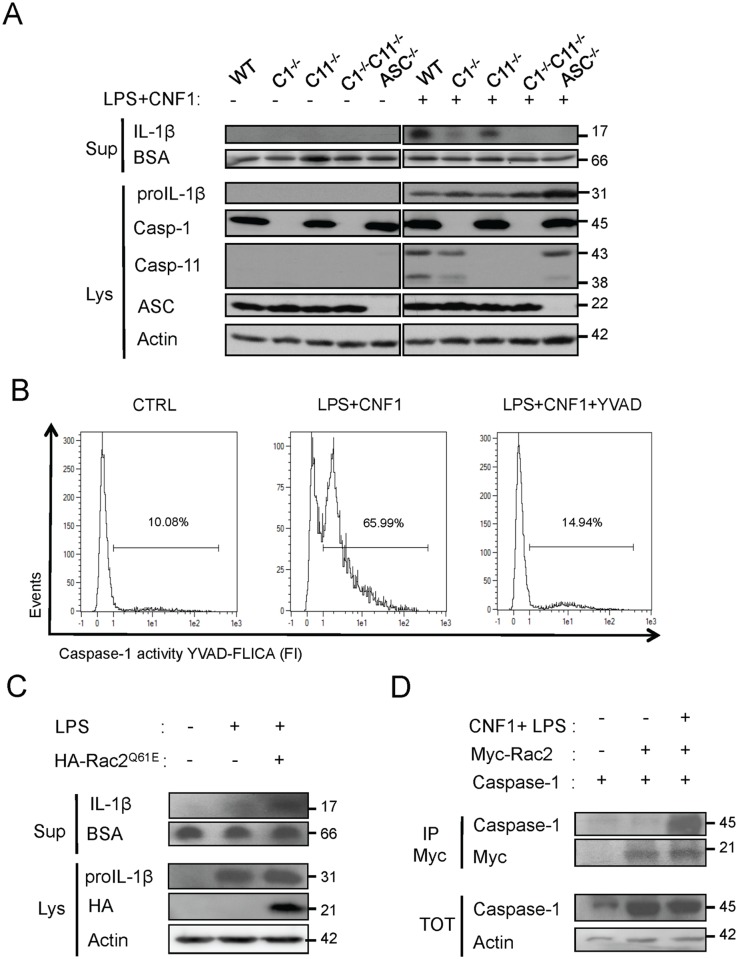
CNF1-triggered IL-1β maturation requires activated Rac, ASC and caspase-1. (A) Western blot analysis of the production and maturation/secretion of IL-1β by primary macrophages following treatment with CNF1, LPS or CNF1+LPS for 10 h. Actin and BSA were used as loading controls. (B) Quantification of caspase-1 activity in macrophages following treatment with CNF1+LPS for 6 h using YVAD-Fluorescent Labelled Inhibitor Caspase-1 Activity (FLICA). (C) Western blot analysis of macrophages IL-1β maturation/secretion upon transfection of HA-Rac2^Q61E^ and LPS treatment. (D) Co-immunoprecipitation of Myc-Rac2 and caspase-1 using an anti-Myc antibody following the treatment of HEK 293T cells with CNF1+LPS for 6 h.

In an approach designed to be complementary to our functional approach, we analyzed the roles of inflammatory caspases in the initiation of the CNF1-dependent innate immune responses during bacteremia. To this end, we measured the levels of the IL-1β and KC cytokines in the sera of infected C1-C11-impaired mice and their congenic wild-type littermates. The wild-type infected with *E*. *coli* ^CNF1+^ displayed higher levels of IL-1β and KC than the WT mice infected with *E*. *coli* ^CNF1-^ (Fig. 3E and F). Interestingly, in the C1–11-impaired mice infected with *E*. *coli* ^CNF1+^, we measured dramatic decreases in the levels of KC and IL-1β in the sera compared with the wild-type mice (Fig. 3E and F). This finding is consistent with the fact that inflammatory caspases-1/11 are critical determinants in the CNF1-triggered cytokine response during bacteremia. Next, we aimed to determine whether the CNF1-triggered IL-1β secretion involved caspase-1 or caspase-11 using bone marrow-derived macrophages isolated from caspase-1 or caspase-11 single knock-out mice. Additionally, we analyzed the effect of CNF1 on macrophages of mice in which the inflammasome adaptor ASC was knocked out. We measured the inhibition of CNF1-triggered IL-1β secretion in macrophages isolated from caspase-1 or ASC but not caspase-11 knock-out mice (Fig. 4A). Thus, our analysis pinpointed the major roles of caspase-1 and ASC in the secretion of IL-1β triggered by CNF1+LPS. In addition, we measured that the activity of caspase-1 increased when monocytes were treated with LPS+CNF1 (Fig. 4B). We then investigated the role of the GTPase Rac in IL-1β secretion triggered by CNF1+LPS. To this aim, we expressed a mutant of Rac2 bearing the modification catalyzed by CNF1 (Rac2^Q61E^). We observed that the expression of Rac2^Q61E^ in the macrophages challenged with LPS promoted the secretion of the p17 form of IL-1β (Fig. 4C). Finally, we were able to detect an association between Rac2 and caspase-1 upon CNF1+LPS treatment specifically by co-immunoprecipitation (Fig. 4D).

Our results identify the Rac/caspase-1/IL-1β signaling axis as a major component of the CNF1-induced elimination of *Escherichia coli* from the bloodstream.

### HlyA counteracts CNF1-triggered immunity

**Fig 5:**
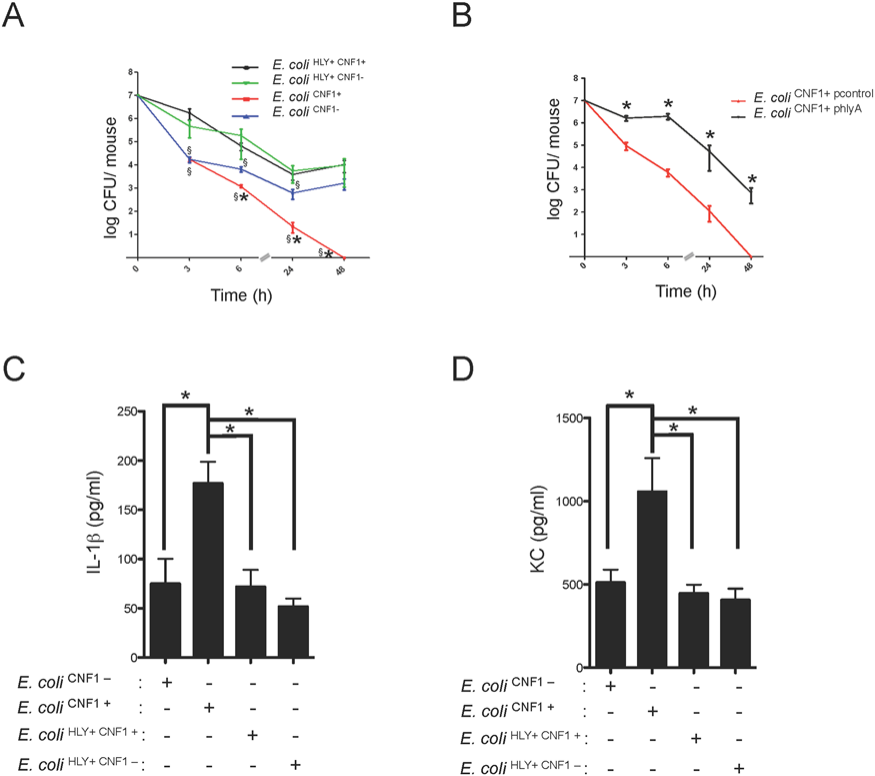
HlyA counteracts the CNF1-induced host response. (A) Female BALB/c mice were intravenously infected with 10^7^ CFUs of *E*. *coli* expressing CNF1 (*E*. *coli* ^CNF1+^) or with 10^7^ CFUs of one of the isogenic mutants, *E*. *coli* ^CNF1-^, *E*. *coli* ^HLY+ CNF1+^ or *E*. *coli* ^HLY+CNF1-^, prior to the collection of peripheral blood at 3, 6, 24 and 48 h for bacteremia measurements. The data are presented as a line graph (n = 20–30; mean ± SEM). P-values<0.05 compared to *E*. *coli* ^CNF1-^ are indicated by (*); and P-values<0.05 compared to *E*. *coli* ^HLY+ CNF1+^ are indicated by (§). (B) Female BALB/c mice were intravenously infected with 10^7^ CFUs of the UTI89Δ*hlyA* strain transformed with a control empty plasmid (*E*. *coli* ^CNF1+ pcontrol^) or with a plasmid encoding HlyA (*E*. *coli* ^CNF1+ phlyA^ *)* prior to the collection of peripheral blood at 3, 6, 24 and 48 h for bacteremia measurements (n = 6–12). (C and D) Analysis of the circulating levels of IL-1β and KC in the sera of C57BL/6 mice were measured by ELISA. Serum samples from mice infected with 10^7^ CFUs of *E*. *coli* ^HLY+CNF1+^ or with 10^7^ CFUs of one of the isogenic mutants, *E*. *coli* ^CNF1+^, *E*. *coli* ^HLY+CNF1-^, or *E*. *coli* ^CNF1-^, were collected at 3 h after intravenous infection and analyzed by ELISA (n = 6). P-values<0.05 (*); and P-values <0.01 (**) were considered statistically significant.

We sought to determine the manner by which pathogenic bacteria cope with anti-virulence immunity. Although HlyA has been shown to interfere with innate immune responses that occur during urinary tract infections, its role during bacteremia is still unknown. We experimentally addressed this question by analyzing the effect of HlyA on CNF1-triggered protection against bacteremia in mice. We observed that all *E*. *coli* strains expressing HlyA displayed increased stability in the blood compared with the other strains, independent of the presence or absence of CNF1 (Fig. 5A). We noticed a reduced bacterial burden in the *E*. *coli* ^CNF1-^ strain compared with *E*. *coli* ^HLY+ CNF1-^. This reduction in the bacterial burden was the greatest in the *E*. *coli* ^CNF1+^ strain. We next complemented the *E*. *coli* ^CNF1+^ strain with a plasmid encoding HlyA (*E*. *coli* ^CNF1+ phlyA^). We found that the complementation of *E*. *coli* ^CNF1+^ with the HlyA expression plasmid stabilized the bacterial load in the blood (Fig. 5B). These results show that HlyA protects *E*. *coli* against host responses, particularly those triggered by CNF1, thereby promoting bacterial stability in the blood.

We went on to determine at which level HlyA acts to prevent CNF1-triggered bacterial clearing. We first investigated whether HlyA has a blocking effect on the activation of the GTPase Rac by CNF1. We detected no modification in the CNF1-mediated activation of Rac when cells were co-incubated with HlyA (S5 Fig). Thus, we hypothesize that HlyA acts downstream of the activation of Rac at the level of cytokine production/secretion. Consistent with this hypothesis, we measured lower levels of IL-1β and KC in the bloodstream of the mice infected with HlyA-expressing *E*. *coli* strains (Fig. 5C and D).

Taken together, our data indicate that HlyA inhibits CNF1-induced pro-inflammatory cytokine responses.

### Gr1^+^ cells are crucial effectors of anti-*E*. *coli* responses in the blood

**Fig 6:**
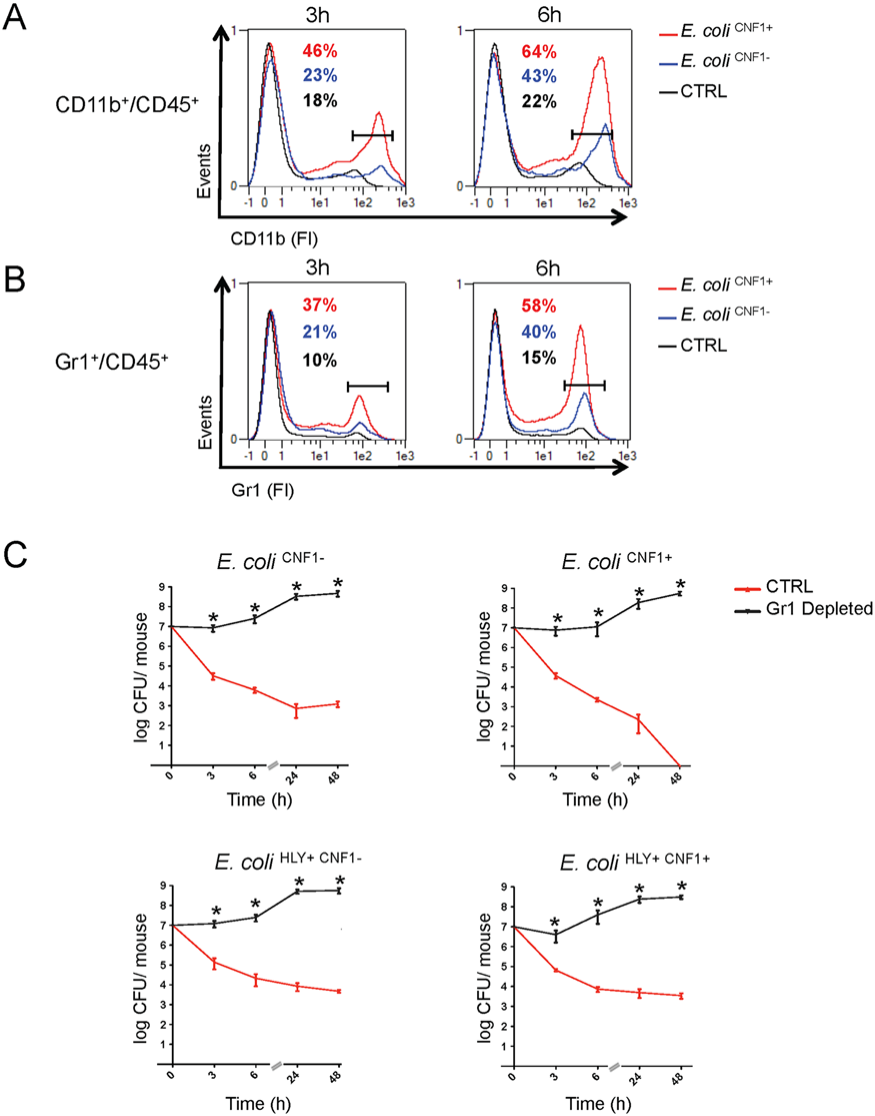
Gr1^+^ cell subpopulations are necessary for the rapid clearance of the CNF1^+^ strain from the blood. (A and B) Flow cytometry analysis of peripheral blood isolated from mice intravenously infected with 10^7^ CFUs of *E*. *coli* ^CNF1+^, *E*. *coli* ^CNF1-^ or PBS (control). Percentages of cells expressing (A) CD45 and CD11b or (B) CD45 and Gr1 are indicated (n = 5). (C) BALB/c mice were injected either with an anti-Gr1 antibody or vehicle, and after 48 h, they were inoculated intravenously with 10^7^ CFUs of *E*. *coli* ^HLY+CNF1+^ or with 10^7^ CFUs of one of the isogenic mutants, *E*. *coli* ^CNF1+^, *E*. *coli* ^HLY+CNF1-^, or *E*. *coli* ^CNF1-^, prior to the collection of peripheral blood at 3, 6, 24 and 48 h for the measurement of CFUs via the plating of serial dilutions. P-values<0.05 (*) were considered statistically significant.

Next, we aimed to further identify the key immune effector cells that control the rapid clearance of *E*. *coli* exacerbated by the Rho-activating toxin CNF1 during bacteremia. We monitored the levels of circulating innate immune cells in the blood at an early time period of infection with either the *E*. *coli* ^CNF1+^ strain or the *E*. *coli* ^CNF1-^ strain. The data were analyzed as the percent of CD45-positive cells, a white blood cell marker, to exclude contamination by red blood cells. We first monitored circulating innate immune cells, including monocytes, neutrophils and granulocytes, using the CD11b marker. Interestingly, we measured higher percentages of CD11b^+^/CD45^+^ cells at both 3 h and 6 h p.i. in the blood of the mice infected with *E*. *coli* CNF1^+^ (3 h: 46% and 6 h: 64%) compared with *E*. *coli* CNF1^-^ (3 h: 23% and 6 h: 43%). Control mice injected with PBS showed lower levels of CD11b^+^/CD45^+^ (3 h: 18% and 6 h: 22%) (Fig. 6A). We found that chemokines, such as MIP1α, MIP1β, MCP-1, and RANTES as well as KC were secreted by the monocytes treated with CNF1 *in vitro* and were increased in the sera of the mice at 3 h p.i. for the *E*. *coli* ^CNF1+^ strain compared with *E*. *coli* ^CNF1-^ (S4B Fig). Because these chemokines are involved in chemotaxis as well as in the activation of neutrophils, we hypothesized that the clearance of the bacteria was due to cooperation between inflammatory monocytes and neutrophils. To test this hypothesis, we monitored the subpopulation of Gr1^+^ cells, including inflammatory monocytes and neutrophils, in the blood of infected mice. Mice infected with *E*. *coli* ^CNF1+^ showed 37% and 58% Gr1^+^CD45^+^ cells at 3 h and 6 h, respectively, compared with only 21% and 40% when the mice were infected with *E*. *coli* ^CNF1-^ (Fig. 6B). These findings indicated that there was an increased recruitment of Gr1^+^ cells triggered by CNF1. The recruitment of Gr1^+^ cells was not reduced in the C1-C11 impaired mice (S6A Fig). Furthermore, the recruitment triggered by CNF1 was not impaired in HlyA-expressing *E*. *coli* (S6B Fig). To demonstrate the key role of Gr1^+^ cells in the clearance of *E*. *coli* expressing CNF1, we depleted this subpopulation, including inflammatory monocytes (Gr1^+^ F4/80^+^) and neutrophils (Gr1^+^ F4/80^-^), prior to infection. We measured an 80% reduction in the Gr1^+^ cell population following the injection of anti-Gr1^+^ (Ly-6G) monoclonal antibodies (RB6–8C5) (S7 Fig). We found that the depletion of Gr1^+^ cells was sufficient to block *E*. *coli* clearance during bacteremia and to prevent the rapid clearance of the *E*. *coli* ^CNF1+^ strain (Fig. 6B).

Altogether, we show that Gr1^+^ recruitment is required but is not sufficient for the anti-virulence immunity triggered by the Rho GTPase-activating toxin CNF1. In addition, we demonstrate that Gr1^+^ cells are critical for the clearance of *E*. *coli* from the bloodstream.

## Discussion

The sensing of the activities of pathogen-encoded virulence factors is emerging as a paradigm of innate immune sensing. However, *in vivo* proof of the contribution of such sensing to mammalian immunity during infection is still scarce. Furthermore, the mechanisms by which pathogenic bacteria cope with the capacities of hosts to detect their virulence remain to be elucidated. As a major discovery, we demonstrated here the capacity of the host to control bacteremia through the exacerbation of LPS-driven IL-1β-mediated antimicrobial responses by CNF1 activity. This host feature relies on Rac/ASC/caspase-1 and the secretion of pro-inflammatory cytokines/chemokines, which in turn mobilize Gr1^+^ cells. Importantly, we described a yet unappreciated role of HlyA in impairing these innate immune responses downstream or in parallel with Gr1^+^ cell recruitment. We also established that pathogen burden and animal death were maximal for the HlyA-positive strains.

Inappropriate, excessive or absent innate immune responses have dramatic consequences on human health. Thus, it is critical to decipher the manner by which the host determines the pathogenic potential of a microbe and responds commensurately [40,41]. It is currently unclear how anti-virulence immunity (AVI) systems of detection work together with the recognition of MAMPs to control inflammation and bacterial virulence. Because CNF1 intoxicates cells without the involvement of additional bacterial factors, it can be used to address this critical question. In this work, we report that the detection of CNF1 activity amplifies the cellular LPS response to a large panel of pro-inflammatory cytokines, including IL-1β, by 2- to 12-fold, thereby producing a more potent immune response. Analysis of the IL-1β level in the sera of mice infected with *E*. *coli* ^CNF1+^ indicated that they exhibited a 3-fold higher response and better resistance to infection than those infected with isogenic *E*. *coli* ^CNF1-^. Our study provides both *in vitro* and *in vivo* evidence that AVI works in concert with MAMP-triggered responses to amplify the innate immune response and ultimately improve host viability. We speculate that this cooperation between AVI and MAMP-triggered immunity is a means by which the host gauges the pathogenic potential of a microbe and tailors a response commensurate with the estimated threat level.

The sensing of the activities of bacterial virulence factors has recently emerged as a conserved means of detecting pathogens. Rho GTPases are targeted by various virulence factors encoded by pathogenic bacteria. These virulence factors either post-translationally modify Rho GTPases by deamidation, glucosylation, adenylylation, or ADP-ribosylation or mimic exchange factors or GTPase-activating proteins, thereby hijacking the GTP/GDP cycle and causing inappropriate activation or inactivation of the critical regulators of these cycles, which include Rho, Rac and Cdc42 GTPases [21]. Interestingly, recent studies have indicated that animal hosts have evolved dedicated strategies for detecting the activities of these virulence factors [26,27,42,43]. Indeed, based on our work focusing on CNF1 and studies of *Salmonella typhimurium* SopE/E2 virulence factors, we can speculate that the abnormal activation of Rac/Cdc42 triggers the assembly of an anti-virulence immune complex involving NOD1 and RIP kinases to promote NF-κB activation and in parallel to assemble a Rac/ASC/caspase-1 complex for the maturation of IL-1β during infections [27,35]. In addition, a recent study has implicated the NLR pyrin as a sensor of the inactivation of the Rho GTPase RhoA by virulence factors via a mechanism that leads to the activation of the pyrin inflammasome and inflammatory caspase-1 [42]. Taken together, these studies indicate that, in parallel with the PRR detection of MAMPS, the host monitors changes in the GTP/GDP cycles of Rho GTPases rather than monitoring each post-translational modification individually, a process that would require a large repertoire of receptors.

Our observation that CNF1 induces an immune response that is detrimental to bacteria raises the question of why CNF1 has been evolutionarily conserved in the UPEC genome. Several reports have established that the CNF1 toxin can trigger the disruption of epithelial cell junctions, promote cell migration and induce the internalization of bacteria into epithelial cells [25]. One hypothesis is that CNF1 has been evolutionarily conserved as an invasion factor to help bacteria cross epithelia during the early stages of infection. Our results led us to speculate that CNF1 has become genetically associated with HlyA to protect the bacteria from an otherwise detrimental CNF1-induced innate immune response. Indeed, the CNF1 and HlyA toxins are co-transcribed within a highly conserved PAI, and epidemiological studies have established that CNF1 is always expressed in association with HlyA [12,19,44,45]. Interestingly, the functional relationship between these toxins described here offers a new framework through which to understand the molecular basis of their tight genetic link and explains why *E*. *coli* that express both of these toxins are pathogenic to mammals. Consistent with this idea, our work sheds light on the manner by which pathogenic bacteria cope with AVI. We report a yet unappreciated role of HlyA in the impairment of innate immune responses. In this role, HlyA has major influences on bacterial burden and host viability. Our genetic analysis revealed that HlyA protects microbes from both CNF1-dependent and CNF1-independent detrimental effects. Mice infected with *E*. *coli* expressing CNF1, but not HlyA, showed an increase in the IL-1β and KC proinflammatory cytokine levels. Given that the bacterial load is minimal under these conditions, this finding cannot be ascribed to increased LPS exposure. It is possible that HlyA targets host immune cell signaling to prevent the production of inflammatory cytokines. This idea is supported by our findings that HlyA did not impair the CNF1-induced activation of Rac, although it blocked the secretion of IL-1β. Further, we showed that bacteria expressing HlyA, but not CNF1, showed an increase in persistence of one log unit in the blood compared with bacteria that were deficient in both toxins. Although HlyA acted primarily to counteract the host recognition of CNF1 activity in our model, it most likely has additional effects on other components of the innate immune response to *E*. *coli*, including phagocytosis or detection by the immune system of other bacterial components [18]. A common feature of pathogenic bacteria is the production of a wide range of pore-forming toxins of various sizes, which have specific ionic and molecular selectivities. It will be important to establish which types of pore-forming toxins are able to block innate immunity and to what extent HlyA blocks the recognition of other factors produced by *E*. *coli*.

Multicellular organisms have evolved sophisticated defense mechanisms to counter microbial attack. In turn, successful microbial pathogens have evolved strategies to overcome host defenses, leading to the occurrence of diseases or chronic infections [46]. In plants, a similar system of detection of the activities of virulence factors has been termed “effector-triggered immunity” [28]. Interestingly, in this model, the pathogen-evolved mechanism counteracting the innate immune defense response has been called a “counter-defense mechanism” [47]. In our model, HlyA counteracts the CNF1-induced host cytokine response. By analogy, if we consider that CNF1 is sensed by the innate immune defense system, HlyA must be considered as a counter-defense effector used by *E*. *coli* to counteract the CNF1-induced host response. The data presented in the present work support a model in which HlyA acts as a major virulence factor that protects microbes from both CNF1-dependent and CNF1-independent innate immune defenses during bacteremia. Based on this model, pore-forming toxins might represent viable drug targets for the treatment of UPEC bacteremia.

## Materials and Methods

### Ethics statement

This study was carried out in strict accordance with the guidelines of the Council of the European Union (Directive 86/609/EEC) regarding the protection of animals used for experimental and other scientific purposes. The protocol was approved by the Institutional Animal Care and Use Committee on the Ethics of Animal Experiments of Nice, France (reference: NCE/2012–64).

### Bacterial strains and toxins

The *E*. *coli* UTI89 clinical isolate was originally obtained from a patient with cystitis [48] and was a kind gift from E. Oswald. The UTI89 streptomycin-resistant (SmR) evolved strain (WT) and isogenic mutants were grown in Luria-Bertani (LB) medium supplemented with streptomycin (200 μg/ml). The CNF1 strain was transformed with a pQE30 plasmid (Qiagen) (*E*. *coli* ^CNF1- pempty^), with pQE30-CNF1 (*E*. *coli* ^CNF1- pcnf1 WT^) or with pQE30-CNF1 C866S (*E*. *coli* ^CNF1- pcnf1 C866S^) and grown in LB supplemented with ampicillin (100 μg/ml) plus IPTG (200 μM) for the infection experiments. The *E*. *coli* ^CNF1+^ strain was transformed with pBR322 (*E*. *coli* ^CNF1+ pcontrol^) or with pEK50 (a plasmid bearing an operon encoding HlyA (*hlyCABD)*) (*E*. *coli* ^CNF1+ phlyA^) and grown in LB supplemented with ampicillin (100 μg/ml). The DH10B K12 *E*. *coli* strain (Life technologies) was transformed with either pEK50 (K12-pHlyA) (a plasmid bearing the operon encoding HlyA (*hlyCABD)*) or the pCR2.1 (K12-pLacZ). The pEK50 plasmid and anti-HlyA antibody were a kind gift from V. Koronakis. The anti-TolC antibody was a kind gift from C. Wandersman. For the infections, a 1/50 dilution of an overnight culture of each strain was inoculated and grown to OD600 = 1.2. Bacteria were either washed in culture medium and diluted to obtain the corresponding MOI for the cell culture infection experiments or were harvested by centrifugation and washed twice in PBS before dilution in PBS to obtain the desired bacterial concentrations for the mouse infection experiments. Recombinant wild-type cytotoxic necrotizing factor-1 (CNF1) and its catalytically inactive form (CNF1-C866S; CNF1 CS) were produced and purified as previously reported [49]. The recombinant proteins were passed through a polymyxin B column (AffinityPak Detoxi-Gel, Pierce). The lack of endotoxin content was verified using a colorimetric LAL assay (LAL QCL-1000, Cambrex). Each stock of the CNF1 preparation (2 mg/ml) was shown to contain less than 0.5 endotoxin units/ml.

### Generation of isogenic bacterial mutant strains

The multi-step procedure used to substitute the *hlyA* and *cnf1* genes in the bacterial chromosome was performed as previously described [50]. Briefly, the pMLM135 plasmid (*cat*, *rpsl*+) was used to transform the UTI89 streptomycin-resistant (SmR) evolved strain. The integration of pMLM135 into the chromosome was selected by plating cells on chloramphenicol-containing medium at 42°C. Excision of the *hlyA* or *cnf1* gene from the chromosome was selected by plating cells on medium containing streptomycin (200 μg/ml). The chromosomal deletions were verified by PCR and by the monitoring of the loss of HlyA and/or CNF1 activity in the deleted strains (S1 Fig). We verified that the isogenic mutant strains had growth properties that were identical to those of the UTI89 strain (S2 Fig). The sequences of the primers used in this study are available upon request.

### Cell lines and primary monocytes

Murine monocytic cells were obtained from pooled blood from 5–10 mice. Monocytes were isolated using a Ficoll-Paque (GE Healthcare) gradient technique. Adherent cells were maintained in M medium [RPMI 1640 medium supplemented with 10% FCS (Lonza), 2 mmol/L L-glutamine, 1 mM pyruvate, 10 mM HEPES, penicillin (100 U/ml), and streptomycin (100 μg/ml)]. When indicated, M-CSF was added as previously described [51]. Cells were transfected using the Amaxa mouse macrophage nucleofector kit according to the manufacturer’s instructions with pCDNA3.1-HA-Rac2Q61E, pRK5-Myc-Rac2, pCAGGS-Caspase-1, pCDNA3.1 or pRK5. Monocyte isolation was confirmed by flow cytometry analysis using F4/80 and CD11b antibodies (Cedarlane). HEp-2 cells and HEK 293T cells were obtained from ATCC (CCL-23 and CRL-3216) and maintained according to ATCC instructions. HEK 293T cells were transfected using Lipofectamine 2000 (Life technologies) according to the manufacturer’s instructions.

### Mouse model of infection

Female BALB/c and C57BL/6 mice (6–8 weeks old) were purchased from Janvier (Le Genest St Isle, France). Caspase-1/11-impaired (also designated as ICE KO) and congenic C57BL/6 mice have been previously described and were kindly provided by R. Flavell [52]. These mice are genetically identical to mice that are now also available from Jackson Laboratories (Stock #016621). Caspase-11 knock-out mice have been previously described by VM Dixit (Genentech). Caspase-1 knock-out mice were generated by D. Dieter and M. Lamkanfy. Their generation will be described elsewhere. Mice were injected i.v. with 10^7^ CFUs of *E*. *coli* as previously described [8,53]. For the determination of bacteremia, blood was collected from the tail vein at the indicated times post-infection, serially diluted in sterile PBS and plated on LB plates containing streptomycin (200 μg/ml) or ampicillin (100 μg/ml) for the strains transformed with pQE30- or the pBR322-derived plasmids, and the plates were incubated for 16 h at 37°C. Injection quality was controlled by plating blood samples obtained from the mice at 5 min after injection. Note that the kinetics for the experiments using the transformed strains were terminated after 24 h because we observed that without selective pressure, the plasmid was stable for up to 24 h. For cytokine analysis, plasma was collected (1200×*g*, 4°C, 5 min) and stored at −20°C.

### *In vivo* Gr1^+^ cell depletion

Mice were injected intraperitoneally with a monoclonal anti-Gr1 antibody (RB6–8C5, 100 μg/20 g body weight). After 48 h, the depletion of Gr1^+^ cells was verified in four mice by analyzing F4/80 and/or Gr1-stained white blood cells by flow cytometry. The anti-Gr1-injected mice were then infected with either UTI89 or UTI89 isogenic mutants.

### Cytokine assays, Caspase-1 assay and antibodies

ELISArrays were performed according to the manufacturer’s instructions (Qiagen, MEM-003A, MEM-004A, MEM-006A, MEM-008A, and MEM-009A). Cytokine concentrations were determined by ELISA and by IL-1β maturation and visualized by western blotting according to the manufacturer’s instructions (KC, TNFα and IL-6, R&D Systems, USA; IL-1β, Raybiotech, USA). Caspase-1 activity was measured using FAM-YVAD-FMK caspase-1 assay kit (ImmunoChemistry Technologies) according to the manufacturer’s instructions. Antibodies used in this study are: anti-IL-1β/IL-1F2 (R&D systems), anti-Caspase-1 (M-20, SantaCruz), anti-Caspase-11 (17D9, Novus), anti-ASC (AL177, Adipogen), anti-HA (16B12, Covance), anti-Myc (9E10, Roche), anti-Rac (102/Rac1, BD Biosciences), anti-GAPDH (FL335, SantaCruz), anti-β-actin (AC-74, Sigma).

### Activated RacGTPase pull-down

Primary monocytes (5x10^6^ cells per condition) were treated with 1 μg/ml of CNF1 toxin for 6 h with or without addition at identical MOI (MOI of 0.5) of live K12 *E*. *coli* expressing HlyA (K12-pHlyA) or LacZ (K12-pLacZ) as a control. Cells were lysed at 4°C using a lysis buffer (Tris 25mM pH 7.5, NaCl 150mM, MgCl_2_ 5mM, EGTA 0.5mM, TritonX100 0.5%, glycerol 4%) and Pull-down assays were performed using 50 μg of GST-PAK^70–106^. Proteins were resolved on 12% SDS-PAGE followed by transfer on PVDF membranes. Equal amount of proteins engaged in the Pull-down assays was confirmed by immunoblotting anti-β-actin.

### Statistical analyses

Statistical analyses were performed using Prism V5.0b software (GraphPad, La Jolla, CA). Unless stated otherwise, comparisons between two groups were performed using the Mann-Whitney nonparametric test, and comparisons among three or more groups were conducted with the Kruskal-Wallis test with Dunn's post-test. P-values<0.05 (*) and P-values<0.01 (**) were considered statistically significant.

## Supporting Information

S1 Fig Genomic and phenotypic characterization of isogenic UTI89 mutants. (A) PCR amplification of bacterial genomic DNA of UTI89 (*E*. *coli* ^HLY+CNF1+^) and the isogenic mutants UTI89Δ*hlyA* (*E*. *coli* ^CNF1+^), UTI89Δ*cnf1 (E*. *coli* ^HLY+CNF1-^), and UTI89Δ*hlyA* Δ*cnf1* (*E*. *coli* ^CNF1-^) using PCR primers specific for the flanking regions of *cnf1* (upper panel) and for the flanking regions of *hlyA* (lower panel) (representative results of n = 3). (B) Hemolytic activity of *E*. *coli* ^HLY+CNF1+^ and the isogenic mutants *E*. *coli* ^CNF1+^, *E*. *coli* ^HLY+CNF1-^, and *E*. *coli* ^CNF1-^ plated on sheep blood agar plates (representative results of n = 3). (C) Bacteria were lysed after 16 h of culture in LB, and the biological activities of CNF1 in *E*. *coli* ^HLY+CNF1+^, the isogenic mutants *E*. *coli* ^CNF1+^, *E*. *coli* ^HLY+CNF1-^, and *E*. *coli* ^CNF1-^, and control untreated cells were assessed after 24 h by HEp-2 multinucleation quantification as previously described [49].
(TIF)

S2 Fig Growth properties and expression of HlyA and CNF1 toxins in the UTI89 strain and in isogenic mutants. (A and B) *E*. *coli* ^HLY+CNF1+^ and the isogenic mutants *E*. *coli* ^CNF1+^, *E*. *coli* ^HLY+CNF1-^, and *E*. *coli* ^CNF1-^ were cultured in LB medium at 37°C. (A) OD600 was measured over time to monitor bacterial growth (n = 3). (B) RNA samples were prepared by TRIzol extraction followed by DNase1 (Qiagen) digestion to prevent DNA contamination. Toxin expression was monitored by qRT-PCR, normalized to 16S RNA and expressed relative to WT UTI89 (n = 3; mean±SD).
(TIF)

S3 Fig ELISArray analysis of the culture supernatants of monocytes treated with CNF1, LPS or with both CNF1 and LPS. Monocytes (5x10^5^ cells per condition) isolated from mouse blood were treated for 10 h with PBS (control) or with 1 μg/ml of CNF1 toxin or with 100 ng/ml LPS or with both 1 μg/ml of CNF1 toxin and 100 ng/ml LPS. The supernatants of the monocyte cultures were analyzed using mouse ELISArray kits. The data are shown as fold inductions compared to the control condition [n = 3; mean (SD)].
(TIF)

S4 Fig Analysis of cytokine secretion triggered by CNF1 *in vitro* and *in vivo*. (A) Cytokine array of monocytes treated with CNF1 or the inactive mutant CNF1C866S. Monocytes (5x10^5^ cells per condition) isolated from mouse blood were treated with 1 μg/ml of CNF1 toxin for 10 h with or without 1 μg/ml of the catalytically inactive CNF1C866S mutant. (B) ELISArray analysis of the sera of mice infected with *E*. *coli* ^CNF1+^ or *E*. *coli* ^CNF1-^. The mouse sera were analyzed using mouse ELISArray kits. The data are shown as fold inductions compared to the control condition [n = 3; mean (SD)]. (C) Primary monocytes (5x10^5^ cells per condition) were treated with PBS (control) or with 0.005 to 5 mM ATP for 10 h with or without 100 ng/ml LPS and compared with primary monocytes (5x10^5^ cells per condition) treated with 0.001 to 1 μg/ml CNF1 toxin for 10 h with or without 100 ng/ml LPS (n = 3). (D) Primary monocytes (5x10^5^ cells per condition) treated for 10 h with Pam3CSK4 or FSL-1 alone or in combination with 1 μg/ml CNF1 toxin or with 1 μg/ml of the catalytically inactive CNF1C866S mutant. IL-1β cytokine secretion was analyzed by ELISA (n = 3).
(TIF)

S5 Fig Impact of the HlyA toxin on CNF1-triggered Rac activation. (A) Primary monocytes (5x10^6^ cells per condition) were treated with 1 μg/ml of CNF1 toxin for 6 h with or without the addition at identical MOI (MOI of 0.5) of live K12 *E*. *coli* transformed with a plasmid bearing the operon encoding HlyA (*hlyCABD)* (K12-pHlyA) or LacZ (K12-pLacZ) as a control. The cells were lysed, and the GTP-bound Rac was isolated using a GST-Pak pull-down assay. Samples were analyzed by immunoblotting. (B and C) *E*. *coli* K12-pLacZ or K12-pHlyA were grown statically in tissue culture media for 6 h. (B) Hemolytic activity of *E*. *coli* K12-pHlyA. Drops of culture of E. coli K12-pLacZ and K12-pHlyA were spotted on sheep blood agar plates and incubated at 37°C for 6 h. (C) Production and secretion of HlyA by K12-pHlyA. *E*. *coli* K12-pLacZ or K12-pHlyA pellets (Pellet) or filtered supernatant (Sup) were analyzed by immunoblotting anti-HlyA toxin (anti-HlyA) and anti- TolC outer membrane protein (anti-TolC) used as a control.
(TIF)

S6 Fig Flow cytometry analysis of the Gr1^+^ and CD11b^+^ populations. (A and B) Flow cytometry analysis of peripheral blood isolated from mice intravenously infected with 10^7^ CFUs of (A) *E*. *coli* ^CNF1+^, *E*. *coli* ^CNF1-^ (B) *E*. *coli* ^CNF1+HLY-^, or *E*. *coli* ^CNF1+HLY+^. The percentages of cells expressing CD45 and CD11b or CD45 and Gr1 are indicated (n = 5).
(TIF)

S7 Fig Flow cytometry analysis of the antibody-triggered depletion of the Gr1^+^ population. Flow cytometry analysis of white blood cells isolated after 48 h by Ficoll-Paque gradient to assess the depletion of the Gr1^+^ population (pooled blood samples from 3 mice).
(TIF)
