# Supplementary figures and images for: Escherichia coli α-Hemolysin Counteracts the Anti-Virulence Innate Immune Response Triggered by the Rho GTPase Activating Toxin CNF1 during Bacteremia
Source: PLoS Pathog. 2015 Mar 17;11(3):e1004732. doi: 10.1371/journal.ppat.1004732 (PMC4363529; doi:10.1371/journal.ppat.1004732)

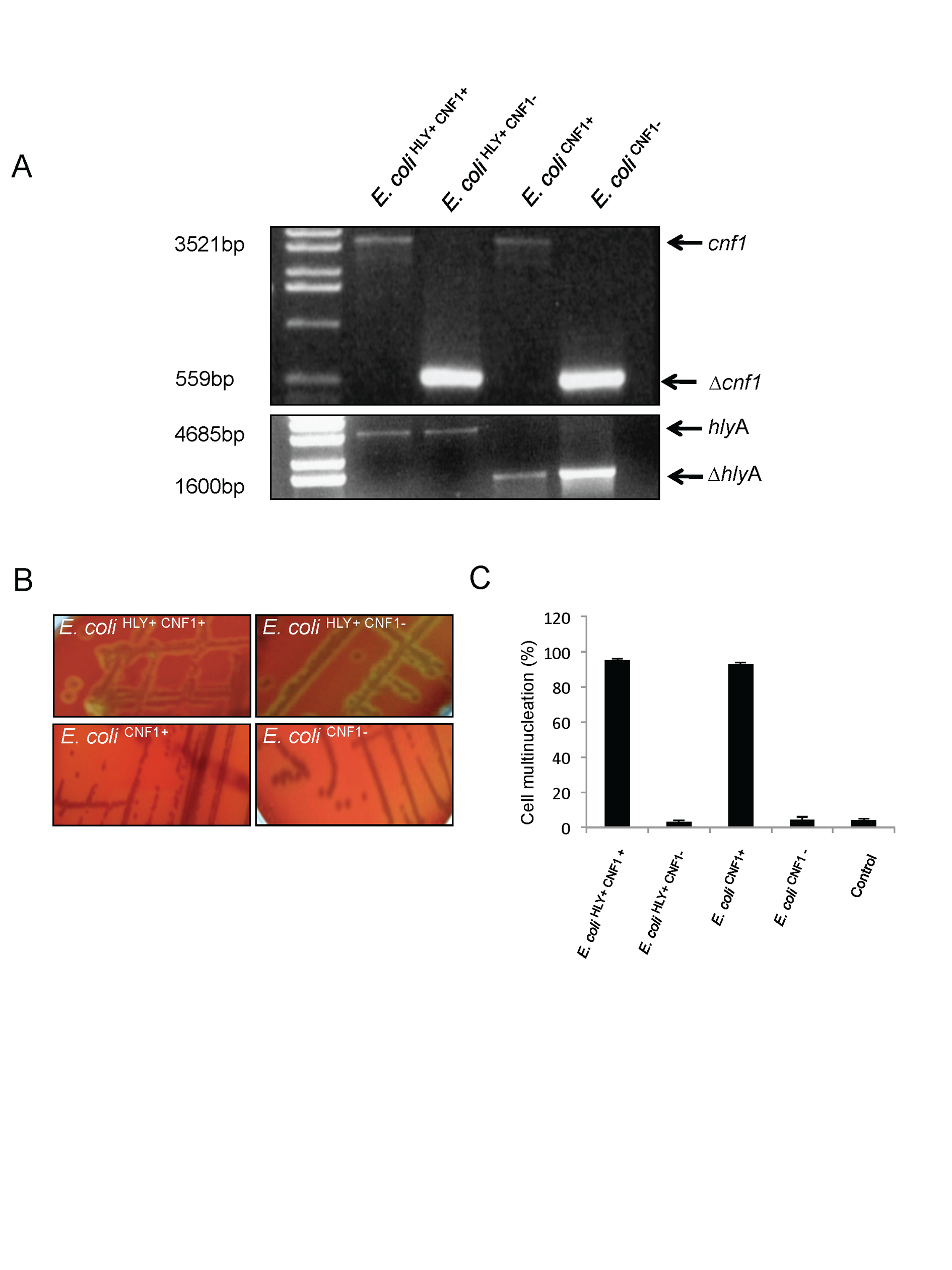

Supplement: S1 Fig — (A) PCR amplification of bacterial genomic DNA of UTI89 (E. coli HLY+CNF1+) and the isogenic mutants UTI89ΔhlyA (E. coli CNF1+), UTI89Δcnf1 (E. coli HLY+CNF1-), and UTI89ΔhlyA Δcnf1 (E. coli CNF1-) using PCR primers specific for the flanking regions of cnf1 (upper panel) and for the flanking regions of hlyA (lower panel) (representative results of n = 3). (B) Hemolytic activity of E. coli HLY+CNF1+ and the isogenic mutants E. coli CNF1+, E. coli HLY+CNF1-, and E. coli CNF1- plated on sheep blood agar plates (representative results of n = 3). (C) Bacteria were lysed after 16 h of culture in LB, and the biological activities of CNF1 in E. coli HLY+CNF1+, the isogenic mutants E. coli CNF1+, E. coli HLY+CNF1-, and E. coli CNF1-, and control untreated cells were assessed after 24 h by HEp-2 multinucleation quantification as previously described [49]. (TIF) [file ppat.1004732.s001.tif]

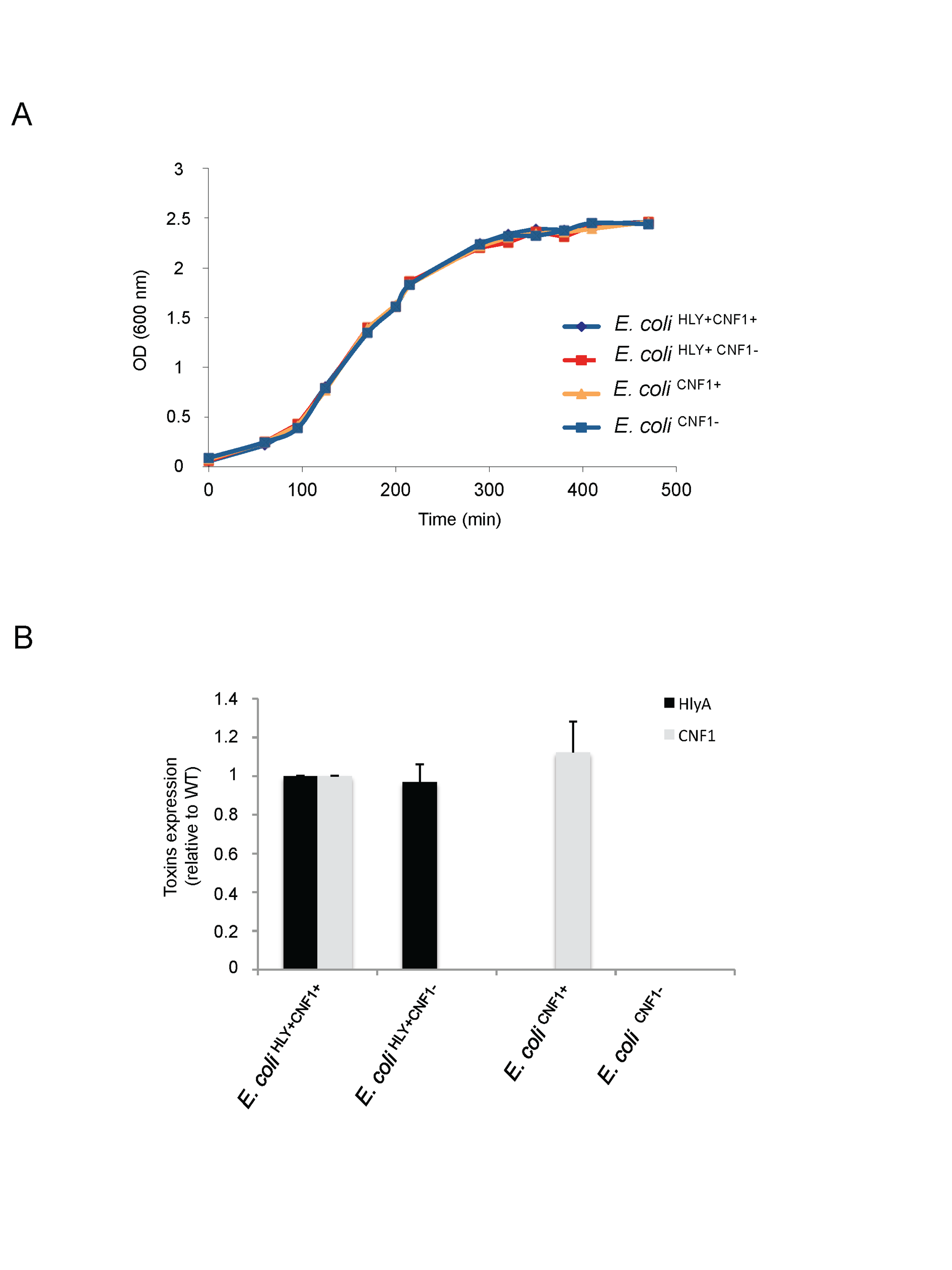

Supplement: S2 Fig — (A and B) E. coli HLY+CNF1+ and the isogenic mutants E. coli CNF1+, E. coli HLY+CNF1-, and E. coli CNF1- were cultured in LB medium at 37°C. (A) OD600 was measured over time to monitor bacterial growth (n = 3). (B) RNA samples were prepared by TRIzol extraction followed by DNase1 (Qiagen) digestion to prevent DNA contamination. Toxin expression was monitored by qRT-PCR, normalized to 16S RNA and expressed relative to WT UTI89 (n = 3; mean±SD). (TIF) [file ppat.1004732.s002.tif]

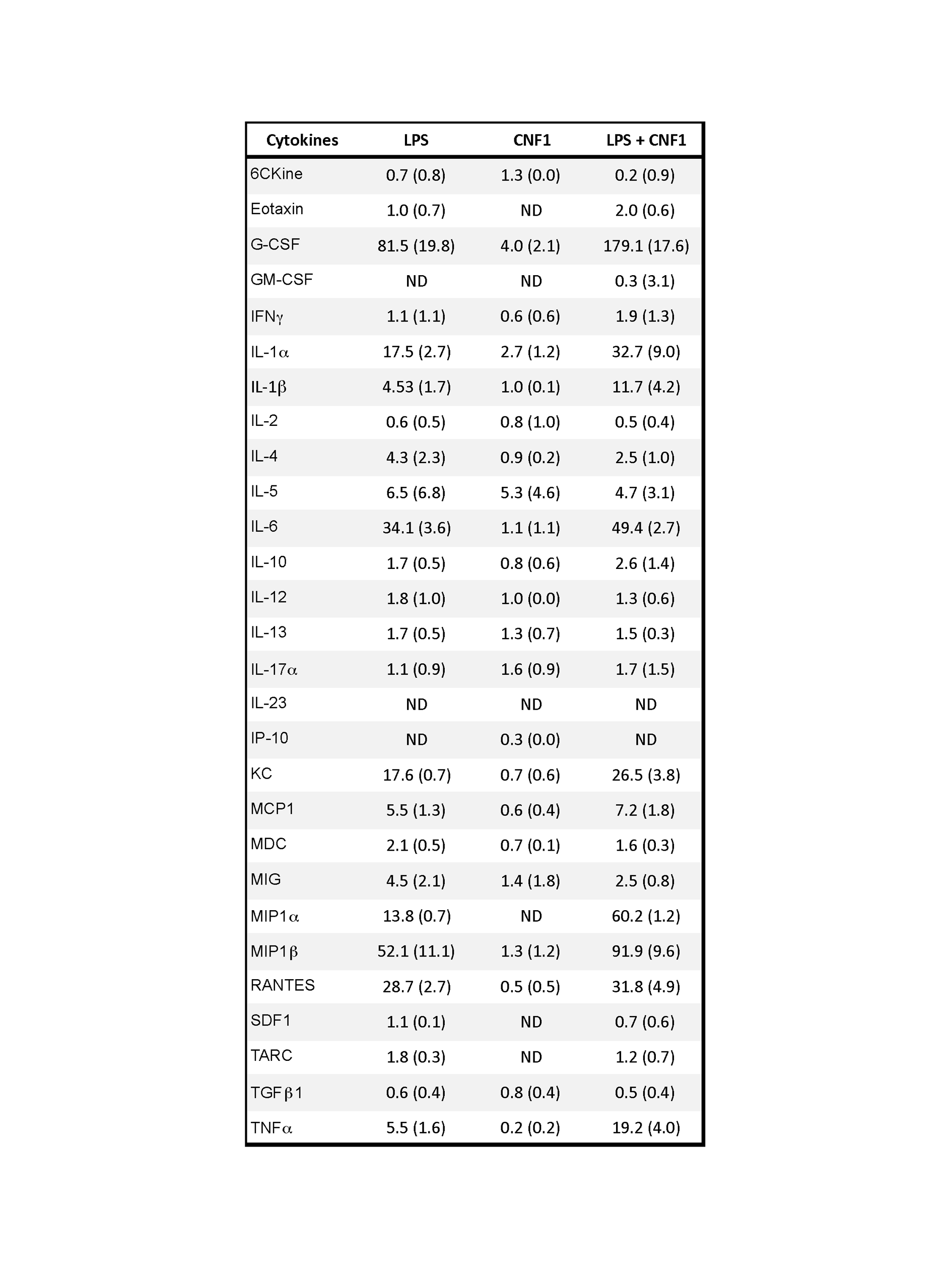

Supplement: S3 Fig — Monocytes (5x10^5^ cells per condition) isolated from mouse blood were treated for 10 h with PBS (control) or with 1 μg/ml of CNF1 toxin or with 100 ng/ml LPS or with both 1 μg/ml of CNF1 toxin and 100 ng/ml LPS. The supernatants of the monocyte cultures were analyzed using mouse ELISArray kits. The data are shown as fold inductions compared to the control condition [n = 3; mean (SD)]. (TIF) [file ppat.1004732.s003.tif]

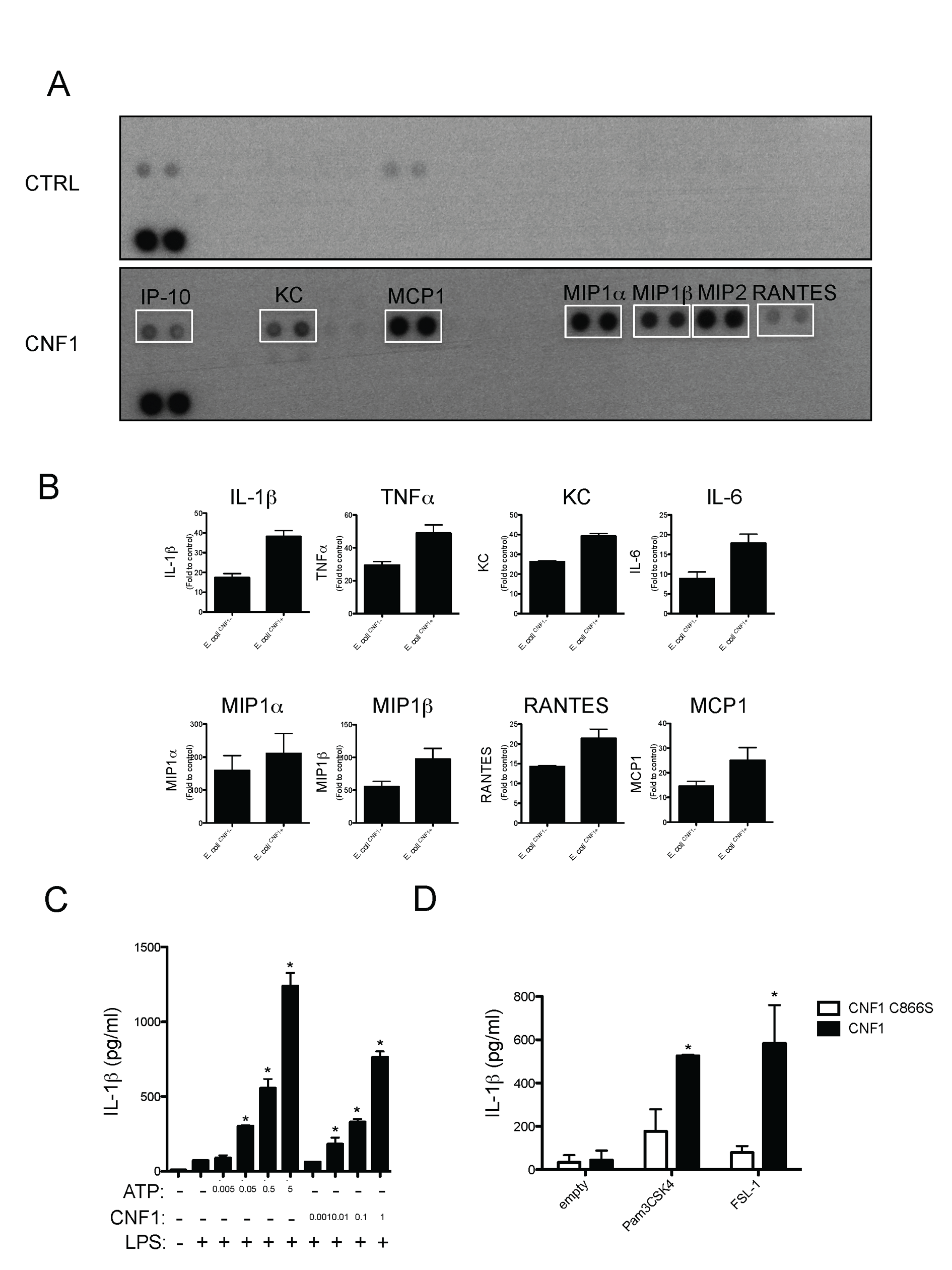

Supplement: S4 Fig — (A) Cytokine array of monocytes treated with CNF1 or the inactive mutant CNF1C866S. Monocytes (5x10^5^ cells per condition) isolated from mouse blood were treated with 1 μg/ml of CNF1 toxin for 10 h with or without 1 μg/ml of the catalytically inactive CNF1C866S mutant. (B) ELISArray analysis of the sera of mice infected with E. coli CNF1+ or E. coli CNF1-. The mouse sera were analyzed using mouse ELISArray kits. The data are shown as fold inductions compared to the control condition [n = 3; mean (SD)]. (C) Primary monocytes (5x10^5^ cells per condition) were treated with PBS (control) or with 0.005 to 5 mM ATP for 10 h with or without 100 ng/ml LPS and compared with primary monocytes (5x10^5^ cells per condition) treated with 0.001 to 1 μg/ml CNF1 toxin for 10 h with or without 100 ng/ml LPS (n = 3). (D) Primary monocytes (5x10^5^ cells per condition) treated for 10 h with Pam3CSK4 or FSL-1 alone or in combination with 1 μg/ml CNF1 toxin or with 1 μg/ml of the catalytically inactive CNF1C866S mutant. IL-1β cytokine secretion was analyzed by ELISA (n = 3). (TIF) [file ppat.1004732.s004.tif]

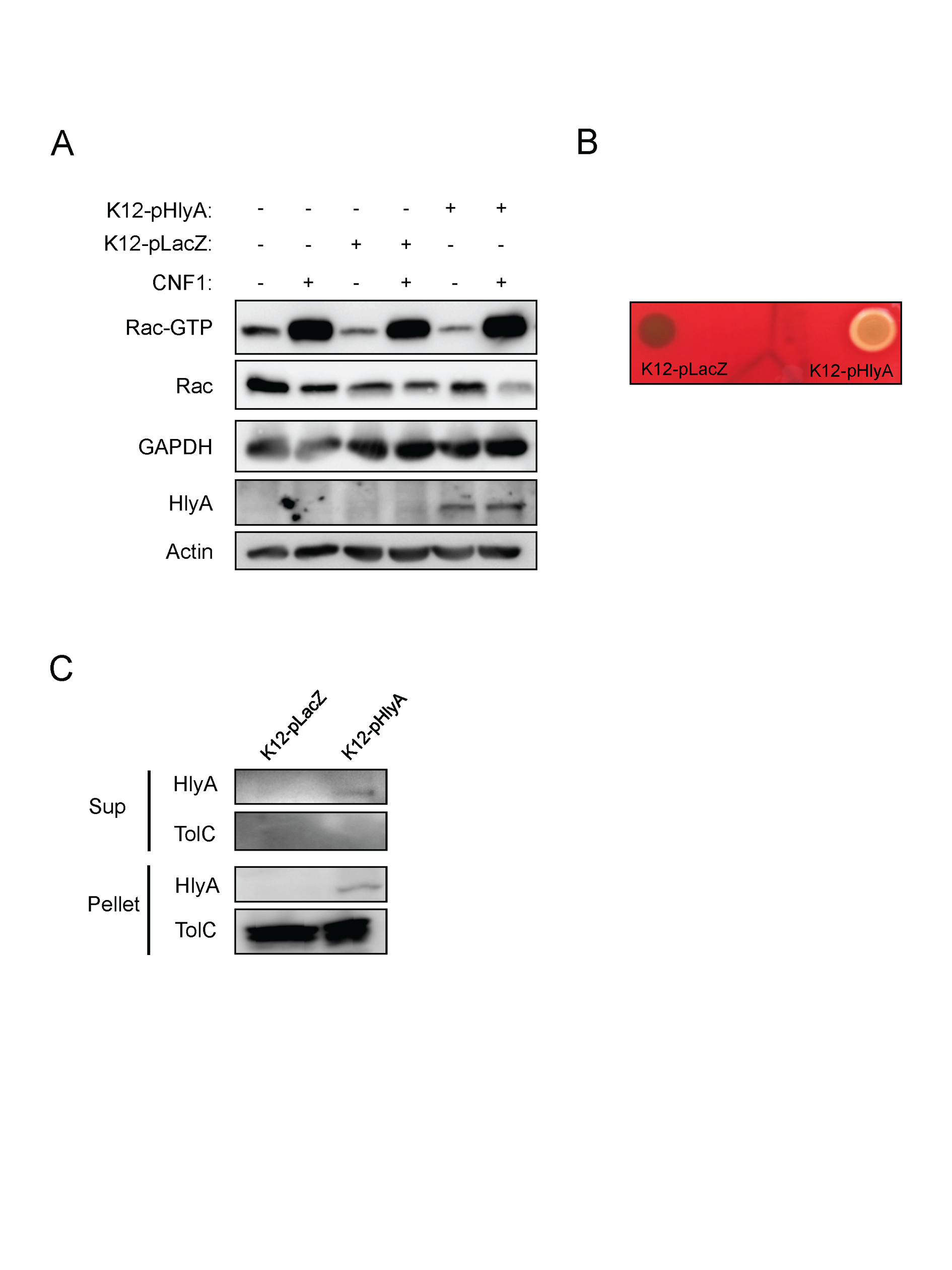

Supplement: S5 Fig — (A) Primary monocytes (5x10^6^ cells per condition) were treated with 1 μg/ml of CNF1 toxin for 6 h with or without the addition at identical MOI (MOI of 0.5) of live K12 E. coli transformed with a plasmid bearing the operon encoding HlyA (hlyCABD) (K12-pHlyA) or LacZ (K12-pLacZ) as a control. The cells were lysed, and the GTP-bound Rac was isolated using a GST-Pak pull-down assay. Samples were analyzed by immunoblotting. (B and C) E. coli K12-pLacZ or K12-pHlyA were grown statically in tissue culture media for 6 h. (B) Hemolytic activity of E. coli K12-pHlyA. Drops of culture of E. coli K12-pLacZ and K12-pHlyA were spotted on sheep blood agar plates and incubated at 37°C for 6 h. (C) Production and secretion of HlyA by K12-pHlyA. E. coli K12-pLacZ or K12-pHlyA pellets (Pellet) or filtered supernatant (Sup) were analyzed by immunoblotting anti-HlyA toxin (anti-HlyA) and anti- TolC outer membrane protein (anti-TolC) used as a control. (TIF) [file ppat.1004732.s005.tif]

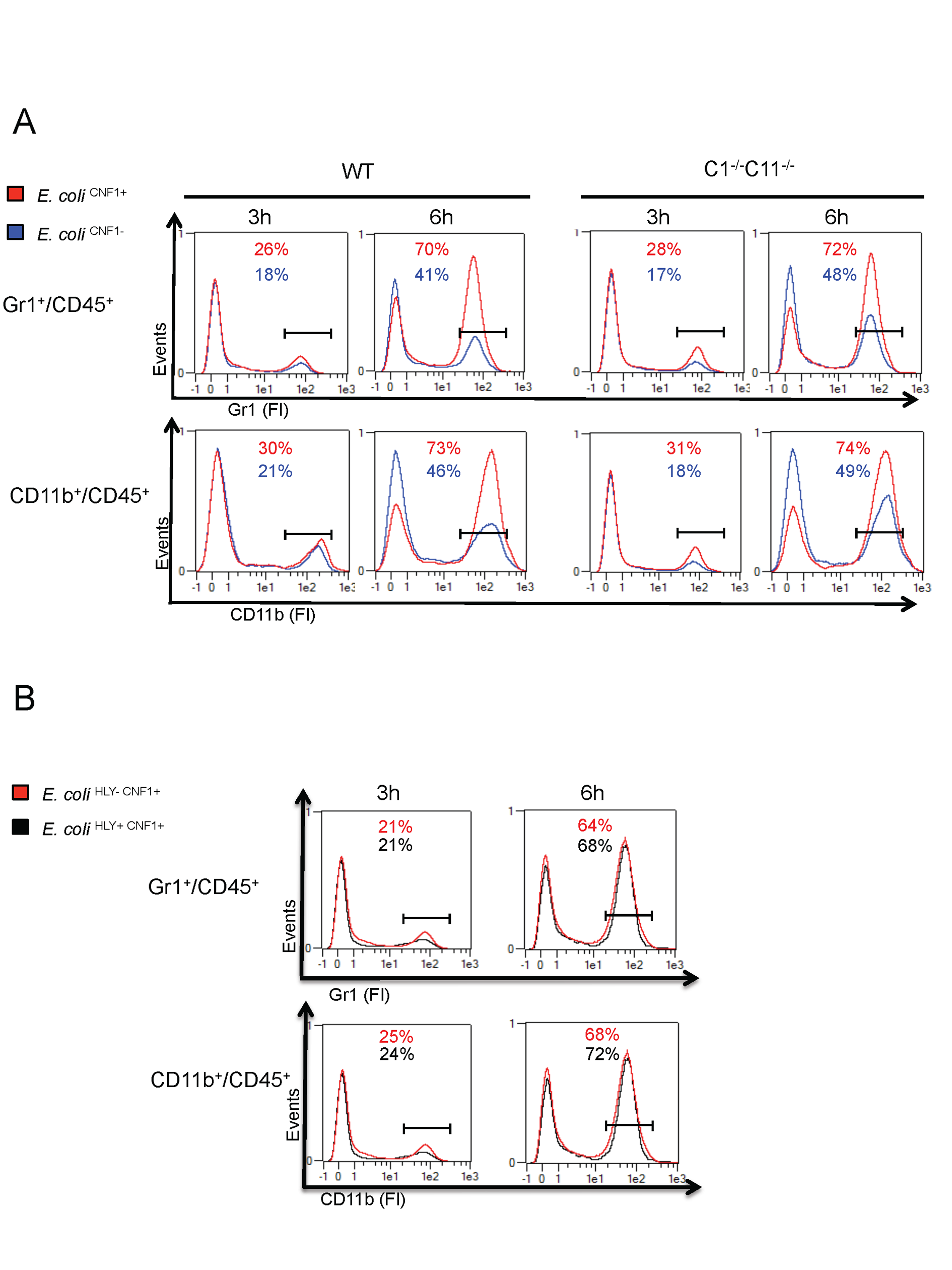

Supplement: S6 Fig — (A and B) Flow cytometry analysis of peripheral blood isolated from mice intravenously infected with 107 CFUs of (A) E. coli CNF1+, E. coli CNF1- (B) E. coli CNF1+HLY-, or E. coli CNF1+HLY+. The percentages of cells expressing CD45 and CD11b or CD45 and Gr1 are indicated (n = 5). (TIF) [file ppat.1004732.s006.tif]

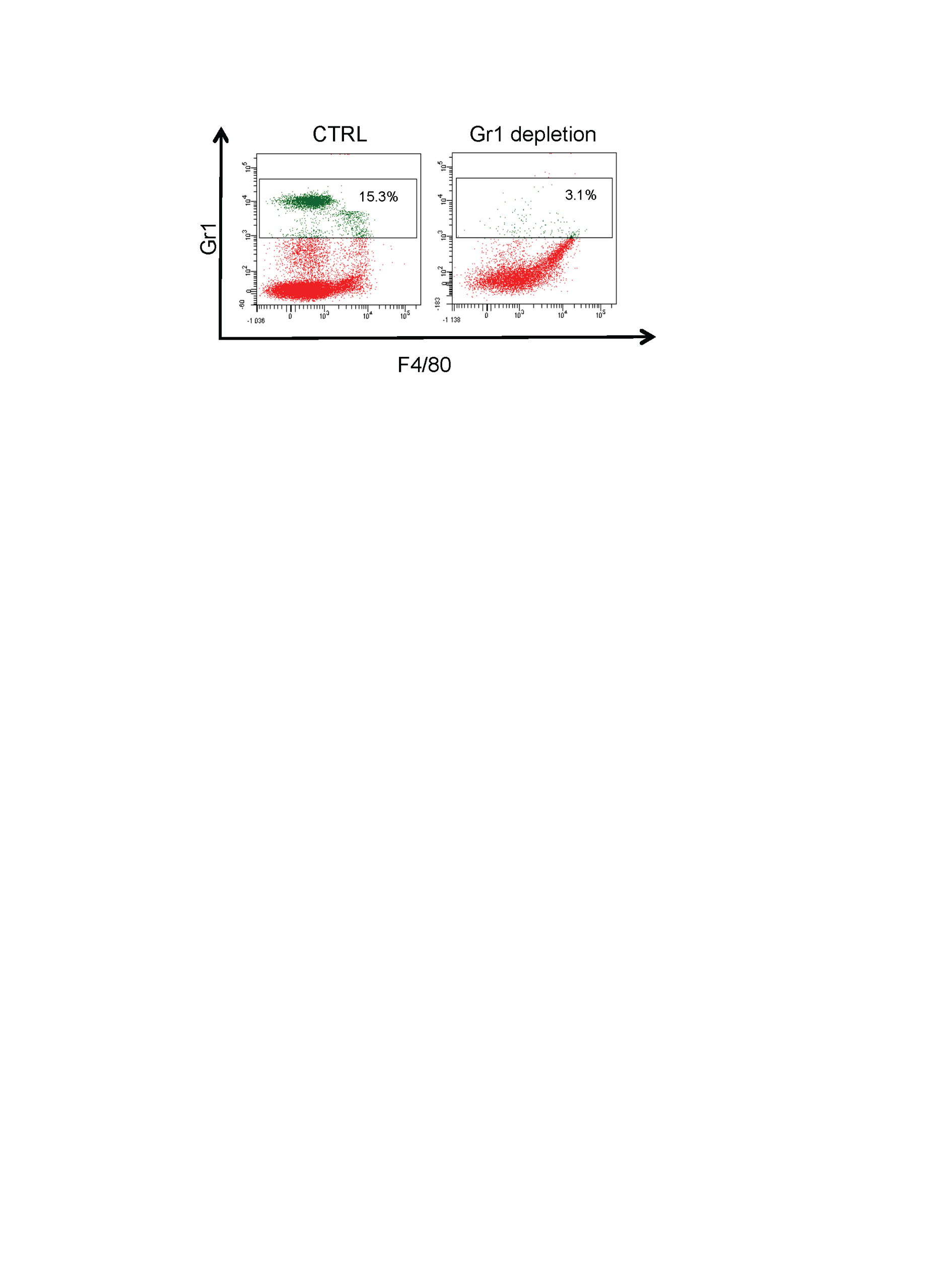

Supplement: S7 Fig — Flow cytometry analysis of white blood cells isolated after 48 h by Ficoll-Paque gradient to assess the depletion of the Gr1+ population (pooled blood samples from 3 mice). (TIF) [file ppat.1004732.s007.tif]
